# MiRNA-Mediated Subpathway Identification and Network Module Analysis to Reveal Prognostic Markers in Human Pancreatic Cancer

**DOI:** 10.3389/fgene.2020.606940

**Published:** 2020-12-09

**Authors:** Yuejuan Liu, Yuxia Cui, Xuefeng Bai, Chenchen Feng, Meng Li, Xiaole Han, Bo Ai, Jian Zhang, Xuecang Li, Junwei Han, Jiang Zhu, Yong Jiang, Qi Pan, Fan Wang, Mingcong Xu, Chunquan Li, Qiuyu Wang

**Affiliations:** ^1^School of Medical Informatics, Harbin Medical University, Daqing, China; ^2^School of Nursing, Harbin Medical University, Daqing, China; ^3^College of Bioinformatics Science and Technology, Harbin Medical University, Harbin, China

**Keywords:** pathway, gene, miRNA, pancreatic cancer, survival

## Abstract

**Background:**

Pancreatic cancer (PC) remains one of the most lethal cancers. In contrast to the steady increase in survival for most cancers, the 5-year survival remains low for PC patients.

**Methods:**

We describe a new pipeline that can be used to identify prognostic molecular biomarkers by identifying miRNA-mediated subpathways associated with PC. These modules were then further extracted from a comprehensive miRNA-gene network (CMGN). An exhaustive survival analysis was performed to estimate the prognostic value of these modules.

**Results:**

We identified 105 miRNA-mediated subpathways associated with PC. Two subpathways within the MAPK signaling and cell cycle pathways were found to be highly related to PC. Of the miRNA-mRNA modules extracted from CMGN, six modules showed good prognostic performance in both independent validated datasets.

**Conclusions:**

Our study provides novel insight into the mechanisms of PC. We inferred that six miRNA-mRNA modules could serve as potential prognostic molecular biomarkers in PC based on the pipeline we proposed.

## Introduction

Pancreatic cancer (PC) is one of the most lethal digestive system tumors characterized by rapid onset, high malignancy and high mortality (Lei et al., 2017). In contrast to the steady increase in survival for most cancers, the 5-year survival of PC patients remains less than 5% (Siegel et al., 2017); however, there has been substantial progress in both diagnostic and therapeutic techniques. Prognosis for surgically treated patients is difficult, and detection of new biomarkers is urgently required in order to accurately predict PC patient outcome and better understand the associated molecular mechanisms.

MicroRNAs (miRNAs) are short, endogenous non-coding RNAs that participate in post-transcriptional gene regulation. Increasing research on PC has revealed that, miRNAs play an important role in the development of PC (Peng et al., 2015; Song et al., 2015; Imamura et al., 2017). Recent literature indicates *miR-339-5p* can suppress the invasion and migration of PC cells via direct regulation of *ZNF689* (Yu et al., 2019). *MiR-137-3p*, as a direct target of *circ-LDLRAD3k* contributed to repressing the proliferation, migration and invasion of PC cells when *circ-LDLRAD3* was downregulated (Yao et al., 2019). Although an increasing number of disease-relevant genes and miRNAs have been identified through microarray and next-generation sequencing, the precise functional mechanism that contributes to the pathology of this complex disease remains unclear.

Pathway analysis is the first choice to gain insight into biological processes and understand the underlying mechanisms of complex diseases. Several studies have shown that subpathways, rather than complete biological pathways, are abnormal in disease phenotypes (Li et al., 2009, 2013). As such, many methods have been developed to identify biological pathways or subpathways (Feng et al., 2016, 2019; Han et al., 2018, 2020; Liu et al., 2019; Ning et al., 2019; Yamaguchi et al., 2019; Li et al., 2020). For example, the Over-Representation Analysis (ORA) method (Breitling et al., 2004) identifies biological pathways by evaluating the extent to which the genes in a gene set of interest appear in given predefined pathway using a hypergeometric test. Another approach is topology enrichment analysis framework (TEAK) (Judeh et al., 2013). TEAK identifies linear and non-linear subpathways and scores them. Emerging evidence suggests that miRNAs play important roles in biological pathways, acting as regulators of pathway output or as important pathway targets (Ooi et al., 2011). Sidiropoulos et al. (2014) found Kallikrein-related peptidase 5 could induce miRNA-mediated anti-oncogenic pathways in breast cancer. Al-Ghezi et al. (2019) demonstrated that combination of Δ9-tetrahydrocannabinol and cannabidiol could induce attenuation of neuroinflammation through miRNA-mediated signaling pathway. A series of studies focused on identifying miRNA-mediated subpathways for deciphering disease mechanisms (Vrahatis et al., 2015, 2016; Ning et al., 2019). The Subpathway-Gmir (Li F. et al., 2015) method identifies miRNA-mediated metabolic subpathways relevant to various diseases by integrating genes of interest, miRNAs, and pathway topologies through building miRNA-regulated metabolic pathways. Vrahatis et al. (2015) developed an effective method for capturing miRNA-mediated signaling subpathways by integrating paired miRNA/mRNA expression data. They subsequently developed time-vaRying enriCHment integrOmics Subpathway aNalysis tOol (CHRONOS) by integrating time series mRNA/miRNA expression data with KEGG pathway maps and miRNA-target interactions (Vrahatis et al., 2016). These studies either focused on identifying miRNA-mediated pathways/subpathways associated with complex diseases, or focused on the impact of miRNA on diseases as regulators or targets of these pathways/subpathways analysis of these pathways/subpathways. However, few studies have analyzed relationships between patient survival and modules extracted from the special network constructed by miRNA-mediated subpathways associated with complex diseases such as PC.

In this study, we propose a novel pipeline that can be used to identify prognostic molecular biomarkers by identifying miRNA-mediated subpathways associated with complex diseases and further extracting modules from a comprehensive miRNA-gene network (CMGN). Briefly, 105 significant miRNA-mediated subpathways were found to be associated with PC. The CMGN was further constructed using these subpathways, and 10 miRNA-mRNA modules were extracted. Functional analyses revealed that the majority of these modules were enriched among cancer-related gene ontology (GO) terms. Finally, an evaluation of the association between survival and the level of gene and miRNA expression in the modules found that six out of the 10 miRNA-mRNA modules were able to discriminate between two groups of PC patients with different clinical outcomes. Together, we provide an effective pipeline for analyzing the relationships of patient survival and modules extracted from the special network constructed by miRNA-mediated subpathways associated with complex diseases. The findings of our study provide novel insight into the mechanisms of PC and identify six modules that may have prognostic significance for predicting the survival of PC patients.

## Materials and Methods

**FIGURE 1:**
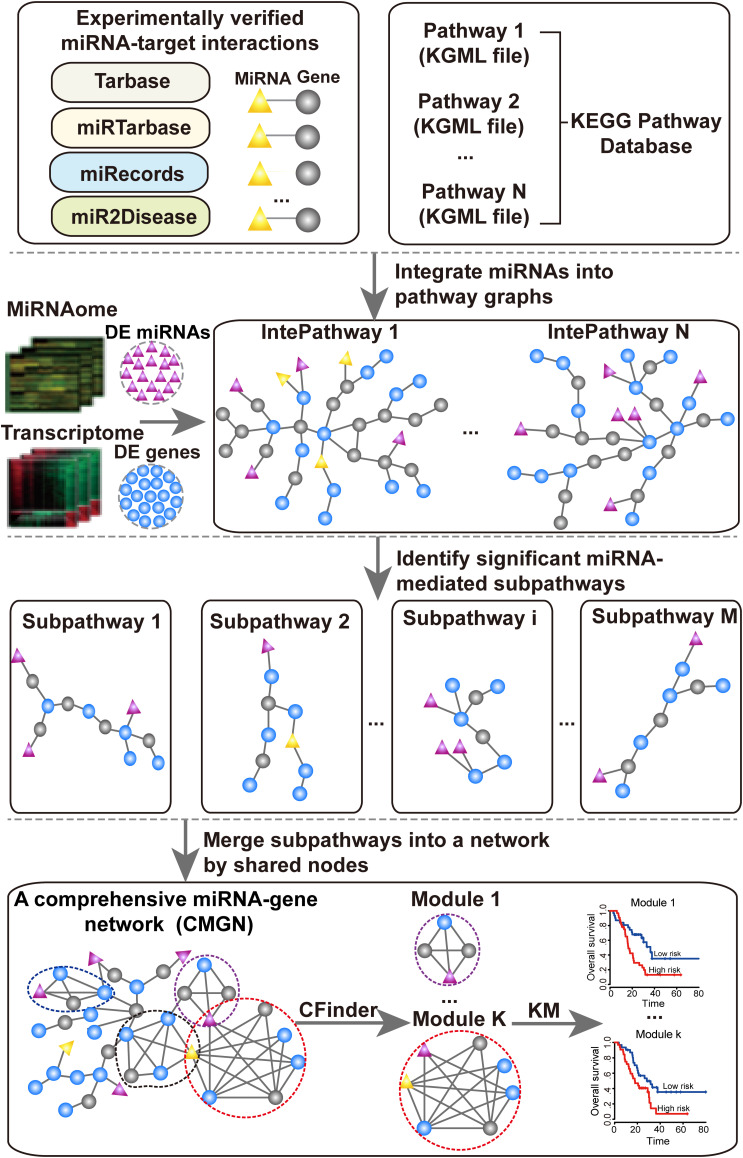
Flow chart of the proposed pipeline.

The aim of this study was to identify significant subpathways associated with PC and evaluate the prognostic value of miRNA-gene modules in the CMGN constructed using these subpathways. Firstly, differentially expressed genes (DE genes) and miRNAs (DE miRNAs) in PC tissues were identified using microarray gene expression profile data of PC. These DE genes and DE miRNAs were subsequently mapped into undirected pathway graphs embedded by miRNA (UPEMs) to detect the miRNA-mediated subpathways associated with PC. The statistically significant subpathways were obtained and defined as PC-relevant subpathways. A comprehensive miRNA-gene network was built by merging common nodes and edges based on the PC-relevant subpathways. Finally, we detected functional modules in the network and further evaluated their value in relation to PC patient survival. An overview of the pipeline is presented in Figure 1.

### PC Dataset

The gene and miRNA expression profiles, including 14 solid-pseudopapillary neoplasms samples, six PC samples, six neuroendocrine tumor samples and five non-neoplastic pancreatic tissue samples, were initially analyzed by Park et al. (2014). We downloaded the expression profile data from the Gene Expression Omnibus (GEO) database (GSE43795 for miRNAs and GSE43796 for mRNAs) (Park et al., 2014). PC and non-neoplastic pancreatic tissue samples were used to identify DE genes and DE miRNAs associated with PC, using the significance analysis of microarrays (SAM) (Tusher et al., 2001) method. An FDR < 0.001 as used for genes and FDR < 0.05 for miRNAs. We identified 1,586 DE genes and 107 DE miRNAs. Additionally, two independent datasets were used to evaluate the prognostic power of the miRNA-mRNA modules. For independent validation dataset 1, a microarray dataset including mRNA expression profiles and clinical information with early stage PC (GSE57495) from 63 patients was downloaded from the GEO database (Edgar et al., 2002). For independent validation dataset 2, an RNA-seq dataset of mRNAs and miRNAs for primary PC tumors, was retrieved from the Cancer Genome Atlas (TCGA) database^1^. The same TCGA barcode structure was used for both clinical data and RNA-seq data. A total of 177 samples with clinical follow-up information were retained for further analysis.

### Experimentally Validated miRNA-Target Interactions

Experimentally validated miRNA-target interaction data were collected from four databases: TarBase (version 6.0) (Vergoulis et al., 2012); miRTarBase (release 2014) (Hsu et al., 2014); mir2Disease (Jiang et al., 2009); and miRecords (release 2013) (Xiao et al., 2009). After removing redundancies, 57,846 pairs of human specific experimentally validated miRNA-target interactions (including 14,614 genes and 579 miRNAs) were obtained. Of these pairs, 12,425 (including 4,347 genes and 371 miRNAs) had been validated by low-throughput experiments.

### Disease-Associated Genes and miRNAs

Disease-associated coding genes were derived from OMIM (Amberger et al., 2011) and GAD (Becker et al., 2004). Disease-associated miRNAs were derived from the miRCancer (Xie et al., 2013), miR2Disease (Jiang et al., 2009), and HMDD (Li et al., 2014) databases. Genes and miRNAs of the significant miRNA-mediated subpathways associated with PC were considered as disease-associated coding genes and miRNAs according to the associations between them and PC recorded in at least one of these databases.

### UPEM Construction

The UPEM was constructed based on KEGG pathways and the experimentally validated miRNA-target interaction data. In this study, 343 KEGG pathways involving 152 metabolic and 191 non-metabolic pathways were used. First, each pathway was converted into an undirected graph with the genes serving as nodes using our previously developed R package “iSubpathwayMiner” (Li et al., 2009). Second, we examined verified targets genes of each miRNA whether appeared in the converted pathway graphs. If its targets were contained in the pathway, we added the miRNA node into the pathway and linked it to their validated targets. Finally, we generated UPEMs that combined the miRNAs and miRNA-target gene interaction edges. We used the 343 pathways embedded by miRNAs to identify the PC risk subpathways.

### Identification of miRNA-Mediated Subpathways and CMGN Construction

The “lenient distance” similarity method (Li et al., 2013; Li F. et al., 2015) was used to identify the miRNA-mediated subpathways in the UPEMs according to coding genes and miRNAs as the input. Notably, the DE miRNAs and DE genes were mapped into UPEMs at the same time as the signature nodes. The shortest path between any two signature nodes in the mapped pathway was computed. Next, the two signature nodes and the molecules contained in the shortest path between them were divided into the same candidate node set, with the requirement that the length of the shortest path be less than or equal to *n*. The corresponding subgraph was extracted according to each candidate node, and set as a subpathway if the node number was no less than *s*. The parameters were set to *n* = 1 and *s* = 10. Statistically significant subpathways were further extracted by performing a hypergeometric test with a *p*-value < 0.001. The *p*-value was calculated according to the following equation:

$$P=1-\sum_{x=0}^{r_{g}+r_{m\,i\,r}-1}\frac{\left(\begin{array}{c} t_{g}+t_{m\,i\,r}\\ x \end{array}\right)\,\left(\begin{array}{c} m_{g}+m_{m\,i\,r}-t_{g}-t_{m\,i\,r}\\ n_{g}+n_{m\,i\,r}-x \end{array}\right)}{\left(\begin{array}{c} m_{g}+m_{m\,i\,r}\\ n_{g}+n_{m\,i\,r} \end{array}\right)}$$

where *m*_*g*_ (*m*_*mir*_) represents the number of coding genes (miRNAs) in the entire human genome, of which *t*_*g*_ (*t*_*mir*_) represents the number of miRNAs involved in a given subpathway, and *n_*g*_ (n_*mir*_)* represents the number of DE genes (DE miRNAs), of which *r_*g*_ (r_*mir*_)* represents the number of DE miRNAs involved in the same subpathway. The CMGN was constructed using all the genes/miRNAs extracted from the statistically significant subpathways and all inherited edges. Finally, for each miRNA in the network, a Pearson correlation between the level of miRNA expression and all genes in the network were computed, and the edges representing the strong negative correlation (*r* < −0.7) between the miRNA and its target genes were specially marked.

### Identification of Disease Related Modules in the miRNA-Gene Network

We extracted the modules from the miRNA-gene network using the Clique Percolation Method (CPM) implemented by CFinder (Adamcsek et al., 2006) software with a value of parameter *k* = 4 (*k*-clique size) for the richest modular structure. CFinder is a program that can rapidly locate and visualize overlapping, densely interconnected groups of nodes in undirected graphs. We interpreted a community constructed from adjacent cliques of the same size *k* in the CPM as a module. A *k*-clique represented a complete subgraph on *k* nodes, and two *k*-cliques were considered to be adjacent if they shared *k−*1 nodes. A community comprised a set of *k*-cliques in which all nodes could be reached via chains of adjacent *k*-cliques from each other.

### GO and Cancer Hallmark Analysis

Gene ontology analyses were performed using an R package clusterProfiler (Yu et al., 2012) for the coding genes of a given module. A *p-value* threshold of 0.01 was used to indicate statistical significance. The GO categories obtained from a previous study (Plaisier et al., 2012) were used as proxies for the characteristic hallmark traits of cancer.

### Functional Analysis of Modules With a Hypergeometric Test

A hypergeometric test was performed on each module to evaluate the extent to which the genes and miRNAs in the module overlapped with the nodes of statistically significant subpathways. The *p-value* was calculated according to the following equation:

$$P=1-\sum_{x=0}^{r-1}\frac{\left(\begin{array}{c} t\\ x \end{array}\right)\,\left(\begin{array}{c} m-t\\ n-x \end{array}\right)}{\left(\begin{array}{c} m\\ n \end{array}\right)}$$

where *m* is the total number of unique nodes in the subpathways located by simultaneously mapping DE miRNAs and DE genes into UPEMs as the signature nodes; *t* is the number of nodes in the chosen subpathway of interest; *n* is the number of nodes in a given module; *r* is the number of common nodes between the chosen subpathway and the given module. A *p-value* threshold of 0.001 was used to indicate statistical significance.

### Survival Analysis

For the specific molecules (genes/miRNAs) in a given module, a univariate Cox regression analysis was carried out to evaluate the association between survival and the expression levels of molecules in the module. A risk score formula was used to evaluate the association between survival and molecule combinations in the given module and calculated using a linear combination of the expression levels of molecules weighted according to their respective Cox regression coefficients from the univariate Cox regression analysis as follows:

$$R\,i\,s\,k\,s\,c\,o\,r\,e=\sum_{i=1}^{n}b_{i}\,E\,x\,p\,\left(i\right)$$

where *b*_*i*_ is the Cox regression coefficient of molecule *i* from the given module, *n* is the number of molecules in the given module, and Exp(*i*) is the expression value of molecule *i* in the corresponding patient. Cancer patients were classified into high and low-risk groups according to the median risk score. For single gene survival prediction, the median expression value of each gene was used as a cut-off to distinguish two groups of PC patients as having either a low or high relative gene expression. A Kaplan–Meier survival analysis was performed for the two patient groups using the R survival package, and statistical significance was assessed using a two-tailed log-rank test. A *p*-value threshold of 0.05 was used to indicate significance.

## Results

### Identification of PC-Relevant Subpathways Mediated by miRNAs

Using the SAM method to test the PC and non-neoplastic pancreatic tissue samples, 1,586 DE genes and 107 DE miRNAs were identified at the FDR level of 0.001 and 0.05, respectively. After mapping these DE genes and DE miRNAs into UPEMs at the same time as signature nodes, 105 significant subpathways were identified at a strict cut-off value of FDR < 0.001 (see section “Materials and Methods”). These significant subpathways, referred to as PC-relevant subpathways, varied from 10 to 85 genes/miRNAs (mean: 24 genes/miRNAs), and were associated with 105 distinct complete pathways (Supplementary Table 1). The top 20 PC-relevant subpathways are listed in Table 1. The coverage rate of known cancer-associated genes and miRNAs were tested for each PC-relevant subpathway. These known cancer-associated genes and miRNAs were derived from the OMIM, GAD, miRCancer, miR2Disease, and HMDD databases. As a result, it was found that each PC-relevant subpathway was associated with an average of 36.7% known cancer-associated genes and miRNAs, some of which even reached 73.7% (Supplementary Table 2).

**TABLE 1:** The top 20 PC-relevant subpathways.

**Sudb pathway ID**	**Pathway name**	**AnnMoleculeRatio**	**AnnBgRatio**	**FDR**
Path:04010_1	MAPK signaling pathway	23/1693	46/31954	0
Path:04110_1	Cell cycle	30/1693	64/31954	0
Path:04151_1	PI3K-Akt signaling pathway	35/1693	62/31954	0
Path:05034_1	Alcoholism	30/1693	52/31954	0
Path:05152_1	Tuberculosis	23/1693	45/31954	0
Path:05164_1	Influenza A	22/1693	41/31954	0
Path:05168_1	Herpes simplex infection	22/1693	39/31954	0
Path:05200_2	Pathways in cancer	38/1693	85/31954	0
Path:05322_1	Systemic lupus erythematosus	19/1693	29/31954	0
Path:04630_1	Jak-STAT signaling pathway	16/1693	22/31954	2.33E-15
Path:04066_1	HIF-1 signaling pathway	20/1693	38/31954	3.18E-15
Path:04020_1	Calcium signaling pathway	18/1693	30/31954	3.89E-15
Path:05166_1	HTLV-I infection	22/1693	49/31954	7.17E-15
Path:05203_2	Viral carcinogenesis	24/1693	61/31954	1.33E-14
Path:04510_1	Focal adhesion	20/1693	43/31954	5.67E-14
Path:04115_1	p53 signaling pathway	17/1693	32/31954	3.28E-13
Path:04060_1	Cytokine-cytokine receptor interaction	15/1693	24/31954	3.52E-13
Path:05161_1	Hepatitis B	21/1693	53/31954	5.16E-13
Path:04810_1	Regulation of actin cytoskeleton	20/1693	49/31954	9.69E-13
Path:04722_1	Neurotrophin signaling pathway	16/1693	30/31954	1.35E-12

**FIGURE 2:**
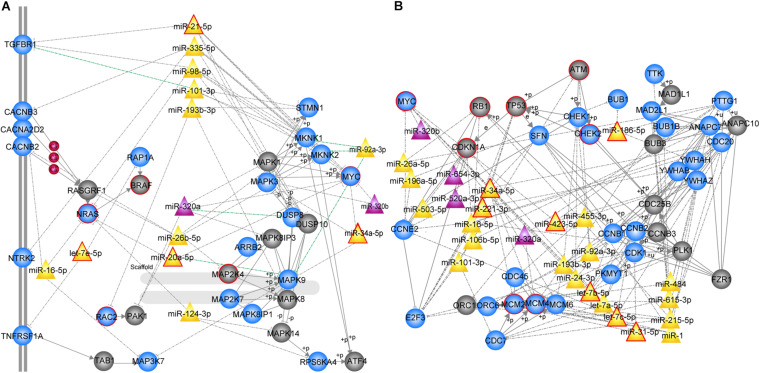
MiRNA-mediated subpathways associated with PC. Circle, triangle nodes represented genes and miRNAs, respectively. DE genes and miRNAs are colored blue and purple, respectively, and non-DE genes and miRNAs are colored gray and yellow, respectively. The red border indicates known PC-associated genes/miRNAs. The edges represented a strong negative correlation (*r* < –0.7) between the miRNA and its target genes marked in green. **(A)** MAPK signaling subpathway (path: 04010_1, *FDR* = 0). **(B)** Cell cycle subpathway (path: 00561_1, *FDR* = 0).

We further focused on the two most significant PC relevant subpathways. The first was path: 04010_1 (FDR = 0), an important sub-region within the MAPK signaling pathway (Figure 2A), which was previously reported to be a highly conserved pathway that transfers extracellular signals into cellular proliferation signals (Aguirre-Ghiso et al., 2003). The mitogen-activated protein kinase (MAPK) is one such complex interconnected signaling cascade with frequent involvement in oncogenesis, tumor progression and drug resistance. One study demonstrated the role of MAPK signaling during the initial steps of pancreatic carcinogenesis (Collins et al., 2014). Based on the topological structure, *miR -320a* was a DE miRNA with a higher degree in this subpathway. Moreover, the over-expression of *miR-320a* strongly contributes to PC pathogenesis, including the characteristics of increased proliferation, invasion, metastasis, drug-resistance and the epithelial-to-mesenchymal transition (Wang et al., 2016). In addition, several DE genes were enriched in this subpathway, among which *MYC*, *NRAS*, *RAC2* were known PC related genes, and most of them play key roles in PC. The study by Adrian et al. suggested that individuals with constitutively decreased *TGFBR1* expression may have a decreased risk of PC (Adrian et al., 2009). *MAPK9* has also been identified as a potentially promising biomarker in exploratory studies of PC (Bracci et al., 2012). More importantly, SNPs in the inflammatory pathway genes *MAPK8IP1* and *SOCS3* were associated with increased overall survival in patients undergoing potentially curative resection and may be used as markers to predict PC patient survival (Reid-Lombardo et al., 2013).

The second significant subpathway was path: 04110_1 (FDR = 0), which is one of the most important cell cycle signaling pathways (Figure 2B) that regulates both cell division and apoptosis. DNA damage readily leads to dysregulation of the cell cycle, which is an essential step in the initiation and development of human cancer (Nan et al., 2016). The 04110_1 subpathway consisted of 64 genes/miRNAs, 15 of which were known PC-associated genes/miRNAs and 30 of which were DE genes/miRNAs. According to the topological structure of the pathway, miRNAs with a higher degree are more important in the subpathway region. It was found that *miR-34a*, a non-differential and disease-associated miRNA, exhibited the highest degree. Moreover, this miRNA has been reported to serve as a diagnostic biomarker for PC (Long et al., 2017). Hu et al. (2013) proposed the use of *miR-34a*-delivering therapeutic nanocomplexes as an effective treatment for PC. Notably, nine target genes of *miR-34a* were identified in this subpathway, including *TP53*, *MYC*, *MCM2*, *MCM4*, *MCM6*, *CDC20*, *SFN*, *CCNE2*, and *E2F3*. Four out of the nine target genes (*TP53*, *MYC*, *MCM2*, and *MCM4*) were known PC-associated genes, and eight were differentially expressed. Among these differentially expressed target genes, cell division cycle 20 (*CDC20*), an anaphase-promoting complex activator, has been observed to be over-expressed in a variety of human cancers and to play an oncogenic role in tumorigenesis (Wang et al., 2015). *E2F3*, as the target gene regulated by *miR-217*, has been shown to be involved in PC cell growth, invasion and apoptosis (Yang et al., 2017). In addition, *miR-215* exhibited the second highest degree and is dysregulated in several human malignancies, including PC. It is speculated that *miR-215* may act as a tumor suppressor in PC, and could serve as a novel therapeutic target for miRNA-based therapy (Li Q.W. et al., 2015). Interestingly, we found that a miRNA set consisting of *miR-92a*, *miR-24, let-7a*, *let-7b*, *let-7c*, *miR-193b*, and *miR-31* was closely correlated with PC. Each of these miRNAs regulates at least one differentially expressed cell cycle-related gene, involving cyclin B1 (*CCNB1*), cyclin B2 (*CCNB2*), and cyclin-dependent kinase 1 (*CDK1*). Among them, *let-7b*, *let-7c*, and *miR-31* are known PC-associated miRNAs, and others have also been associated with PC in previous studies (Listing et al., 2015; Xiao et al., 2017). Our results demonstrate that the PC-relevant subpathways identified by integrating DE genes, DE miRNAs and pathway topologies were closely related to PC.

### Global Properties of the CMGN

**FIGURE 3:**
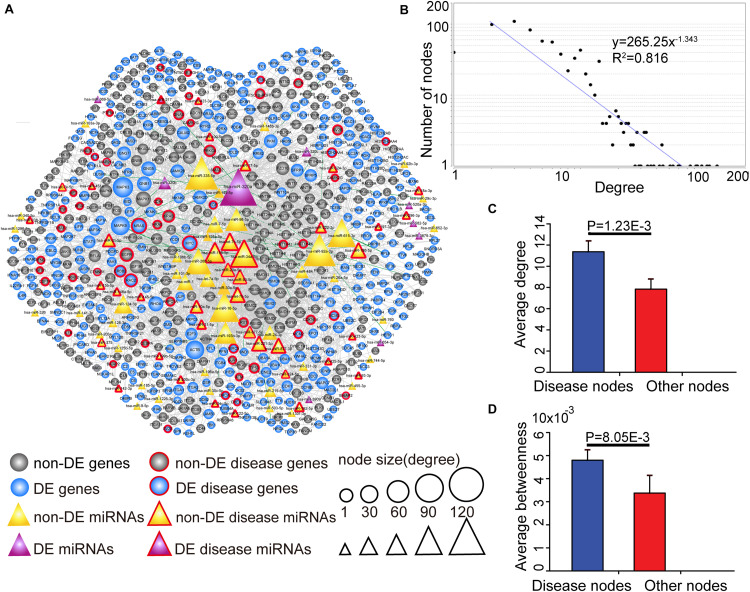
The view and topological features of the CMGN. **(A)** The view of the CMGN. The CMGN consisted of 640 genes and 91 miRNAs with 3,030 edges between them. Of these, 48 edges represented a strong negative correlation (*r* < –0.7) between the miRNA and its target genes marked in green. Disease-associated genes/miRNAs in the CMGN exhibited specific topological characteristics compared to the other nodes. **(B)** The network revealed a power law distribution with a slope of –1.343 and *R*^2^ = 0.816. The *X*-axis indicates the degree of node distribution. The *Y*-axis indicates the number of nodes according to the *X*-axis. **(C)** and **(D)** Disease-associated nodes had a higher degree and betweenness centrality than the other nodes. Data are shown as the mean ± SEM. Disease-associated and other nodes are indicated in red and blue along the *X*-axis. The average degrees of the two groups of nodes are indicated by the *Y*-axis.

Since key genes and miRNAs might participate in multiple subpathways, the CMGN was constructed by merging common nodes and edges of PC-relevant subpathways (see section “Materials and Methods”). In the network, there were 3,030 edges between 91 miRNA nodes and 640 gene nodes, of which 48 edges had a strong negative correlation between the level of miRNA and target gene expression (*r* < −0.7) (Figure 3A). We evaluated the degree of node distribution in the network and observed a power-law and exponential distribution, respectively (Figure 3B). Therefore, the CMGN displayed scale-free characteristics, indicating that it was not random but organized according to a core set of principles in its structure that distinguished it from randomly linked networks (Barabasi et al., 2011).

The known PC-associated miRNAs and genes were further mapped to the network, and 93 PC-associated molecules (39 known PC-associated miRNAs and 54 known PC-associated genes) were found to be involved in the network. The topological characteristics of the network were examined, revealing that these known PC-associated miRNAs and genes showed a significantly higher degree and betweenness centrality than the other miRNAs and genes (Figures 3C,D). The specific topological patterns reflected the functional importance of the known PC-associated miRNAs and genes in the CMGN. A higher degree indicated that the nodes were likely to be hubs and had high probabilities of engaging in essentially biological functions. A higher betweenness centrality implied that they acted as bridges connecting different network components and controlling communication.

All of the nodes’ topological features of the network were ranked and the top 10 genes and miRNAs of each dimension are listed in Table 2. It was observed that all top 10 genes were differentially expressed, three of which (*NRAS*, *MYC*, and *EGFR*) have been well-described as known PC-associated genes. *MAPK3* (also known as *ERK1/2*) is a member of the MAP kinase family that exhibited the highest degree, and appeared in 55 PC-relevant subpathways. MAPK3 is activated by upstream kinases, which results in its translocation to the nucleus where it phosphorylates nuclear targets. Moreover, *MAPK3* plays an important role in the MAPK/ERK cascade. Indeed, *MAPK3* inhibitors can inhibit the growth of PC cells through the RAS-RAF-MEK-ERK pathway (Walters et al., 2013). *MAPK9* exhibited the second highest degree and was inversely correlated with the expression of seven miRNAs in our study, including four known PC-associated miRNAs (*miR-93*, *miR-20a*, *miR-17*, and *miR-320a*). *MAPK9* has previously been identified as a potentially promising biomarker in exploratory studies, and was observed to be overexpressed in PC patients (300 cases, 300 controls) (Bracci et al., 2012). In addition, *miR-320a* had the highest degree among miRNAs and appeared in 92 PC-relevant subpathways. The study by Wang et al. (2016) found that *miR-320a* over-expression promoted PC cell proliferation, migration and invasion, and demonstrated that *miR-320a* suppressed *PDCD4* mRNA expression in 5-Fluorouracil-resistant human PC cells. These findings suggest that *miR-320a* may serve as a potential target for PC therapy (Wang et al., 2016). When applied to clinical serum samples, *miR-320a* could accurately predict late chronic pancreatitis (Xin et al., 2017). Together, these results demonstrate that the CMGN based on PC-relevant subpathways can provide insight into cancer-associated transcriptional regulatory networks. Thus, the CMGN may be able to identify key factors that participate in multiple pathways.

**TABLE 2:** The top 10 genes/miRNAs with high degree and betweenness.

**Gene**	**Degree**	**Gene**	**Betweenness**	**MiRNA**	**Degree**	**MiRNA**	**Betweenness**
MAPK3	51	MAPK9	0.05485797	miR-320a	120	miR-320a	0.16631591
MAPK9	45	MAPK3	0.04162308	miR-335-5p	104	miR-335-5p	0.15280337
ACTB	44	MYC	0.03048808	miR-92a-3p	100	miR-92a-3p	0.08100672
NRAS	34	ACTB	0.03012864	miR-16-5p	91	miR-16-5p	0.06466747
MYC	32	NRAS	0.02392137	miR-615-3p	84	miR-34a-5p	0.063773
MAPK1	30	RHOA	0.02192821	miR-34a-5p	82	miR-615-3p	0.05594317
RHOA	29	EGFR	0.01991883	miR-26b-5p	74	miR-26b-5p	0.04664383
GNB1	28	IGF1R	0.0190645	miR-98-5p	61	miR-155-5p	0.04227725
GNG5	28	RXRB	0.01895021	miR-193b-3p	59	miR-124-3p	0.04179475
EGFR	26	PKM	0.01830244	miR-155-5p	54	miR-193b-3p	0.03668304

### The Identification of Key Functional miRNA-mRNA Modules in the CMGN

**FIGURE 4:**
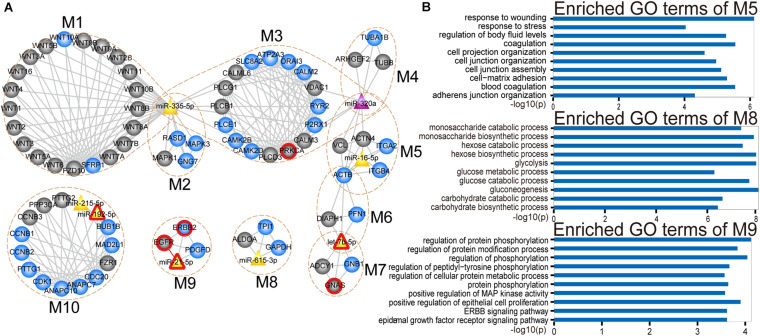
Modular analysis of the CMGN. **(A)** A total of 10 miRNA-mRNA modules were derived from the CMGN, ranging between 4 and 23 nodes, with an average of 8.1 nodes per module. The modules are encircled with an ochre dotted oval. The circle and triangle nodes represent genes and miRNAs, respectively. DE genes and miRNAs are colored blue and purple, and non-DE genes and miRNAs are colored gray and yellow, respectively. Disease associated genes/miRNAs are indicated with red circles. **(B)** GO terms enriched for modules M5, M8, and M10.

Analysis of the CMGN can provide a global view of the regulatory relationships involved in PC-relevant subpathways. To reveal the detailed modular organization of the CMGN, the functional modules in the network were mined using the Clique Percolation Method implemented by CFinder software with a value of parameter *k* = 4 (see section “Materials and Methods”). A total of 30 modules were detected, among which greater attention was paid to the miRNA-mRNA modules. There were 12 modules that contained at least one miRNA and mRNA, two of which were absolutely included in the other modules. Therefore, the larger modules were retained. Ultimately, 10 non-redundant miRNA-mRNA modules were obtained, consisting of 4 ∼ 23 genes/miRNAs (Figure 4A).

For each miRNA-mRNA module, a GO analysis of the coding genes from a given module was carried out based on the GO terms (see section “Materials and Methods”), and each module was annotated with the enriched functions of the corresponding gene set (Supplementary Figure 1). Processes for the maintenance of cell homeostasis (e.g., cell cycle regulation and cytosolic calcium ion homeostasis) and cancer development-related processes (e.g., cell-matrix adhesion and JAK-STAT cascade and glycolysis) were highly represented. Cancer is a complex disease characterized by select hallmarks of cancer, including resistance to cell death, tissue invasion and metastasis, as well as the induction of angiogenesis (Hanahan and Weinberg, 2011). These hallmarks provide a framework for understanding the remarkable diversity of various cancers. Thus, through comparing the GO categories used as proxies for hallmark cancer traits (see section “Materials and Methods”), we found that modules M1, M5, M8, and M10 were involved in five hallmark traits, including positive regulation of signal transduction, cell adhesion, glycolysis and positive regulation of cell growth. Module M5 was enriched in cell-matrix adhesion and mesodermal cell differentiation. Module M8 was enriched in canonical glycolysis and glycolytic processes. Module M9 was enriched in cell proliferation related processes (Figure 4B).

We tested the extent to which the molecules overlap between each miRNA-mRNA module and PC-relevant subpathways using a hypergeometric test with a *p-value* < 0.0001 and annotation proportion ≥ 70% (see section “Materials and Methods”). As a result, nine out of 10 miRNA-mRNA modules were annotated in at least one PC-relevant subpathway (Supplementary Table 3). We found that the modules, that overlapped with other modules, were enriched in more PC-relevant subpathways, indicating that these modules may have multiple functions. For instance, module M5, which overlapped with M3, M4, and M6, was enriched in six PC-relevant subpathways: (1) path: 04510_1 that belonged to focal adhesion; (2) path: 05410_1 that belonged to hypertrophic cardiomyopathy; (3) path: 05414_1 that belonged to Dilated cardiomyopathy; (4) path: 05412_1 that belonged to arrhythmogenic right ventricular cardiomyopathy; (5) path: 04810_1 that belonged to regulation of actin cytoskeleton; and (6) path: 04520_1 that belonged to adherens junction. Interestingly, three of these subpathways enriched by module M5 were associated with cardiomyopathy. To our knowledge, advanced cancer can induce fundamental changes in metabolism and promote cardiac atrophy and heart failure. Thackeray et al. (2017) discovered systemic insulin deficiency in cachectic cancer patients and demonstrated that cancer-induced systemic insulin depletion contributes to cardiac wasting and failure. In addition, low-dose insulin supplementation was found to attenuate these processes in mice with advanced melanoma or colon carcinoma.

Module M9 contained four molecules (*ERBB2*, *EGFR*, *PDGFD*, and *miR-21*), three of which are known PC-associated genes and miRNAs. Studies have shown that increasing *EGFR* activity can over-activate downstream pro-oncogenic signaling pathways (e.g., RAS-RAF-MEK-ERK MAPK, and AKT-PI3K-mTOR pathways), which can then activate many biological outputs that may contribute to cancer cell proliferation (Wee and Wang, 2017). *EGFR* was also identified as a hub in the CMGN, indicating that *EGFR* is able to crosstalk with other molecules. Studies have demonstrated that a shorter *EGFR* intron 1 CA repeat length is associated with a worse PC clinical prognosis (Tzeng et al., 2007). ERBB2, also known as HER2, was functionally characterized by an extraordinarily strong catalytic kinase activity, and represents a key oncoprotein that can trigger cornerstone intracellular signaling events for cell growth and survival, further leading to increased signal transduction and activation of the MAPK and PI3K/Akt pathways (Wong and Hurvitz, 2014). Moreover, Cheng et al. (2017) identified the *ERBB2* exon17 mutation as an independent factor associated with overall survival among metastatic PC patients. Dillhoff et al. (2008) demonstrated that *miR-21* was significantly over-expressed in PC. Although we could not find any direct evidence to support an important role of *PDGFD* in PC, we found that *PDGFD* could regulate many cellular processes, including cell proliferation, apoptosis, transformation, migration, invasion, angiogenesis and metastasis (Wang et al., 2010). Recent studies (Walters et al., 2013; Ogawa et al., 2015) have also shown that *PDGFD* is closely related to various cancers.

### miRNA-mRNA Modules Are Potential Prognostic Biomarkers for PC

**FIGURE 5:**
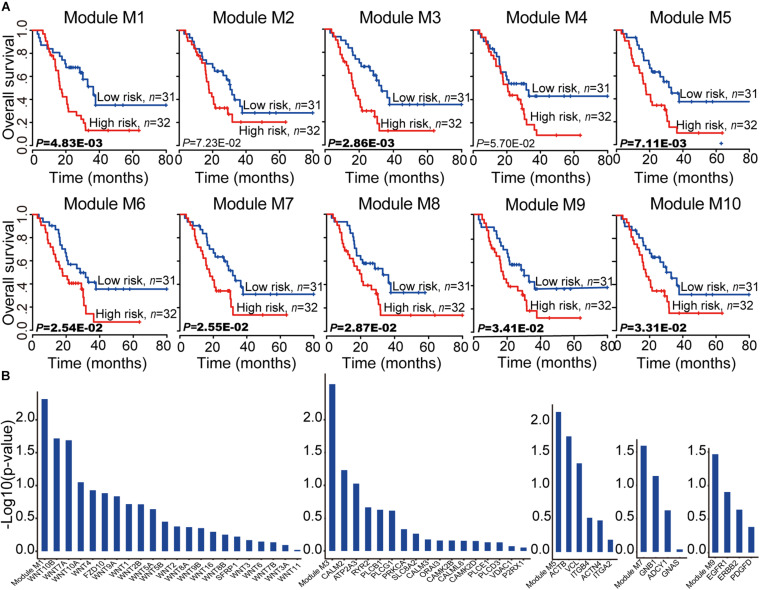
Survival analyses of PC patients for miRNA-mRNA modules. **(A)** Kaplan–Meier survival analyses of all patients with clinical follow-up information based on the 10 miRNA-mRNA modules. Survival months are shown along the *X*-axis. The overall survival rates are shown along the *Y*-axis. A *p*-value threshold of 0.05 was used. **(B)** The miRNA-mRNA module as a whole could better distinguish the two groups of patients compared to any individual coding gene within it, especially modules M3, M7, and M9. The whole module and associated coding genes are presented along the *X*-axis. The log value (base 10) of the overall survival rates are shown along the *Y*-axis.

To assess the prognostic performance of these miRNA-mRNA modules, a survival analysis was performed on each of the 10 miRNA-mRNA modules. After testing all the coding genes of the given module with the risk score model (see section “Materials and Methods”), eight out of 10 modules were found to be significantly related to the prognosis in independent validation dataset 1 (Figure 5A). In each of the eight modules, the patients in independent validation dataset 1 were divided into either a high-risk score or low-risk score group. Patients in the high-risk-score group had a significantly shorter overall survival than those in the low-risk score group. To test whether the whole miRNA-mRNA module showed better prognostic performance than a single coding gene, we calculated the Kaplan–Meier *p-value* of every single coding gene of the modules in independent validation dataset 1. Five out of eight modules (module M1, M3, M5, M7, and M9) showed better prognostic performance than any single coding gene in the module (Figure 5B). More interesting, each coding gene of module M3, M7, and M9 was almost non-significantly related to prognosis; however, the three modules were all significantly related to the prognosis in independent validation dataset 1. For example, each coding gene of module M3 showed bad prognostic performance according to non-significant Kaplan–Meier *p-value.* But the whole miRNA-mRNA module of M3 showed better prognostic performance. Another survival analysis was also carried out on each of the 28 non-redundant modules to test whether their expression levels were associated with PC prognosis. A total of 17 out of 28 non-redundant modules were related to the prognosis of the PC patients based on the level of gene expression. Furthermore, 13 out of 17 modules showed better prognostic performance with the whole module than any individual coding gene (Supplementary Table 4). These findings indicate that the miRNA-mRNA modules in the CMGN provide clinical guidance for cancer prognosis.

To test the reproducibility of the 10 miRNA-mRNA modules’ prognostic performance, all coding genes of the given module in independent validation dataset 2 were tested using the same model and criteria as validation dataset 1. Two modules (modules M1 and M4) were excluded due to a matching of the genes to the mRNA expression profiles of less than 60% (Supplementary Figure 2). Seven out of eight modules (M2, M3, M5, M6, M8, M9, and M10) were related with the prognosis of the PC patients. Six out of seven modules (M3, M5, M6, M8, M9, and M10) showed good prognostic performance in both validation datasets 1 and 2, indicating good reproducibility. To test whether the combination of the miRNAs and mRNAs could predict the survival of PC patients, the relationship of the PC patients’ outcome and 10 miRNA-mRNA modules were further evaluated both coding genes and miRNAs using based on the same model and criteria. We observed a smaller Kaplan–Meier *p-value* based on the level of coding gene and miRNA expression levels compared to only the level of coding gene expression (Supplementary Figure 3). These findings indicate that the majority of miRNA-mRNA modules exhibit good reproducibility, and the involvement of miRNA expression levels may improve the prognostic performance of these modules for PC patients. Furthermore, the six good reproducible modules could serve as potential prognostic molecular biomarkers in PC.

Since the module consisted of connected genes and miRNAs in the network, there may be redundant genes/miRNAs in the module for predicting the survival of PC patients. To select the best prognostic signature, we compared the performances of all the gene combinations in each good reproducible module. A survival analysis was subsequently performed on every combination of genes in each good reproducible module using the same model as independent validation dataset 1. Considering the Kaplan–Meier *p-value* of all combinations in each module, one of these selected combinations was defined as the best signature. The most significant molecule combinations of the six good reproducible modules are listed in the Supplementary Tables 5–10. As expected, four out of six good reproducible modules (modules M3, M5, M9, and M10) exhibited the best signature, which could be represented by fewer *molecules*, for predicting the PC patients’ survival. For example, module M5 could be used to separate PC patients into high and low-risk groups using all genes and miRNAs to a greater extent than any single coding gene. In addition, a combination of two genes (*ITGB4* and *VCL*) was associated with a better prognostic performance. Studies have shown that high levels of *ITGB4* expression are significantly correlated with the hallmarks of epithelial-mesenchymal transition, high tumor grade, and the presence of lymph node metastasis, and also exhibit an independent prognostic effect (Masugi et al., 2015). Moreover, *VCL* has been identified as a potential novel oncogene in pancreatic adenocarcinoma (Loukopoulos et al., 2007). Taken together, four modules (modules M3, M5, M9, and M10) among the six good reproducible modules were found to play an important role for the prognosis of PC patients.

## Discussion

The dysregulation of miRNA expression has been widely observed in the development and progression of complex human diseases, such as cancer. In this study, DE genes and miRNAs were mapped into undirected pathway graphs embedded by miRNA as signature nodes. We obtained 105 significantly miRNA-mediated subpathways associated with PC as PC-related subpathways using a hypergeometric test. The PC-related subpathways provide biological insight for dissecting PC pathology. The key genes or miRNAs may participate in multiple pathways. Therefore, a comprehensive miRNA-gene network was built by merging common nodes and edges. Next, the topological characteristics of the network were analyzed and functional modules were detected. Finally, the functions of modules containing miRNAs were analyzed and their effect on the outcome of PC patients was evaluated. As a result, 105 subpathways were identified to be significantly associated with PC, in which more than a third of the nodes were found to be known cancer-associated genes and miRNAs. We focused on two PC-relevant subpathways belonging to the MAPK signaling pathway and cell cycle. The network analysis of the CMGN built by the PC-related subpathways revealed that the known PC-associated *genes/miRNAs* showed a significantly higher degree and betweenness centrality than other the nodes, indicating their functional importance. A total of 30 modules were detected in the CMGN using CFinder software, of which 10 miRNA-mRNA modules were further examined. A GO enrichment analysis revealed that the miRNA-mRNA modules were highly enriched in processes involved in the maintenance of cell homeostasis and cancer development. The survival analysis was performed on 10 miRNA-mRNA modules in two independent validation datasets. Of these, six of the modules showed good prognostic performance in both validation dataset 1 and 2. In particular, while each coding gene of modules M3, M7, and M9 was not significantly related with prognosis, the three modules were all significantly related to prognosis in independent validation dataset 1. These results revealed that the six good reproducible modules could serve as potential prognostic molecular biomarkers in PC.

The findings of our study provide a novel pipeline that can be used to identify prognostic molecular biomarkers from a comprehensive miRNA-gene network based on the identified miRNA-mediated subpathways associated with PC. However, the regulation between miRNA and mRNA is complex. The expression of miRNAs or mRNAs might be affected by other ncRNAs, including circRNAs and lncRNAs. For example, Zhao et al. (2017) demonstrated that *lncRNA TUG1* could competitively sponge *miR-382*, thereby regulating *EZH2*. Their experiments further revealed that *TUG1* overexpression promoted cellular proliferation and migration, and contributed to epithelial-mesenchymal transition (EMT) formation in PC cell lines (Zhao et al., 2017). Similar studies have demonstrated that *lncRNA H19* could partially promote PC cell invasion and migration by increasing HMGA2-mediated EMT through antagonizing *let-7* in PC cell lines (Ma et al., 2014). *Hsa_circ_0005785* was down-regulated in 20 sets of PC tissues and was inferred to interact with *miR-181a* and *miR-181b* based on the sequence analysis. *MiR-181a* plays an important role in regulating PC cell growth and migration and *miR-181b* has been shown to be related to PC cell resistance to gemcitabine (Li et al., 2016). Identification of the upstream regulatory pathways of miRNAs contributes to research into the functional of miRNAs in various diseases. Information about the regulatory elements involved in the regulation of miRNA transcription is stored in recently published databases, including ENdb (Bai et al., 2020) and SEdb (Jiang et al., 2019). Moreover, the information associated with these regulatory elements (Qian et al., 2019) can be integrated to construct a miRNA transcription regulatory network for a specific disease. Finally, the pipeline we proposed can work flexibly in practices. It supports other methods for identifying differentially expressed subpathways in complex diseases. Similarly, it also supports other methods for mining modules in the network formed by identified subpathways. In parallel, the pipeline is suitable for analyzing integrated networks and can be applied to other complex diseases.

## Data Availability Statement

The datasets generated for this study can be found in online repositories. The names of the repository/repositories and accession number(s) can be found in the article/Supplementary Material.

## Author Contributions

YL analyzed data and wrote the manuscript. YL and YC collected the datasets. XH, YJ, and ML participated in the pre-processing of the datasets. XB, CF, ML, and JnZ performed the computational analysis. YC, JgZ, JH, BA, XL, JH, QP, FW, and MX performed the literature validation. CL and QW conceived the idea for the manuscript, provided guidance and critically revised the manuscript. All authors read and approved the final version to be published.

## Conflict of Interest

The authors declare that the research was conducted in the absence of any commercial or financial relationships that could be construed as a potential conflict of interest.

## Footnotes

1 http://tcga-data.nci.nih.gov/tcga/

## Supplementary Material

Supplementary Figure 1 The enriched GO terms for miRNA-mRNA modules.

Supplementary Figure 2 Survival analysis for miRNA-mRNA modules based on genes.

Supplementary Figure 3 Survival analyses for miRNA-mRNA modules based on miRNAs and genes.

Supplementary Table 1 PC-relevant subpathway list.

Supplementary Table 2 Known cancer-associated genes and miRNAs inPC-relevant subpathways.

Supplementary Table 3 Enrichment for modules on PC-relevant subpathways.

Supplementary Table 4 Survival analysis for modules and individual genes.

Supplementary Table 5 An exhaustive survival analysis for module M3.

Supplementary Table 6 An exhaustive survival analysis for module M5.

Supplementary Table 7 An exhaustive survival analysis for module M6.

Supplementary Table 8 An exhaustive survival analysis for module M8.

Supplementary Table 9 An exhaustive survival analysis for module M9.

Supplementary Table 10 An exhaustive survival analysis for module M10.

## References

[B1] Adamcsek B. Palla G. Farkas I. J. Derenyi I. Vicsek T. (2006). CFinder: locating cliques and overlapping modules in biological networks. *Bioinformatics* 22 1021–1023. 10.1093/bioinformatics/btl039 16473872

[B2] Adrian K. Strouch M. J. Z0eng Q. Barron M. R. Cheon E. C. Honasoge A. (2009). Tgfbr1 haploinsufficiency inhibits the development of murine mutant Kras-induced pancreatic precancer. *Cancer Res.* 69:9169. 10.1158/0008-5472.can-09-1705 19951995

[B3] Aguirre-Ghiso J. A. Estrada Y. Liu D. Ossowski L. (2003). ERK(MAPK) activity as a determinant of tumor growth and dormancy; regulation by p38(SAPK). *Cancer Res.* 63 1684–1695. 12670923

[B4] Al-Ghezi Z. Z. Miranda K. Nagarkatti M. Nagarkatti P. S. (2019). Combination of Cannabinoids, Delta9- Tetrahydrocannabinol and Cannabidiol, ameliorates experimental multiple sclerosis by suppressing neuroinflammation through regulation of miRNA-mediated signaling pathways. *Front. Immunol.* 10:1921. 10.3389/fimmu.2019.01921 31497013 PMC6712515

[B5] Amberger J. Bocchini C. Hamosh A. (2011). A new face and new challenges for online mendelian inheritance in man (OMIMÂ^®^). *Hum. Mutat.* 32 564–567. 10.1002/humu.21466 21472891

[B6] Bai X. Shi S. Ai B. Jiang Y. Liu Y. Han X. (2020). ENdb: a manually curated database of experimentally supported enhancers for human and mouse. *Nucleic Acids Res.* 48 D51–D57. 10.1093/nar/gkz973 31665430 PMC7145688

[B7] Barabasi A. L. Gulbahce N. Loscalzo J. (2011). Network medicine: a network-based approach to human disease. *Nat. Rev. Genet.* 12 56–68. 10.1038/nrg2918 21164525 PMC3140052

[B8] Becker K. G. Barnes K. C. Bright T. J. Wang S. A. (2004). The genetic association database. *Nat. Genet.* 36 431–432. 10.1007/978-3-319-20883-1_17 15118671

[B9] Bracci P. M. Mi Z. Young S. Wiemels J. (2012). Serum autoantibodies to pancreatic cancer antigens as biomarkers of pancreas cancer in a San Francisco Bay Area case-control study. *Cancer* 118 5384–5394. 10.1002/cncr.27538 22517435 PMC3414682

[B10] Breitling R. Amtmann A. Herzyk P. (2004). Iterative Group Analysis (iGA): a simple tool to enhance sensitivity and facilitate interpretation of microarray experiments. *BMC Bioinform.* 5:34. 10.1186/1471-2105-5-34 15050037 PMC403636

[B11] Cheng H. Liu C. Jiang J. Luo G. Lu Y. Jin K. (2017). Analysis of ctDNA to predict prognosis and monitor treatment responses in metastatic pancreatic cancer patients. *Intern. J. Cancer* 140:2344. 10.1002/ijc.30650 28205231

[B12] Collins M. A. Yan W. Seboltleopold J. S. Pasca D. M. M. (2014). MAPK signaling is required for dedifferentiation of acinar cells and development of pancreatic intraepithelial neoplasia in mice. *Gastroenterology* 146 822–834.e7. 24315826 10.1053/j.gastro.2013.11.052 PMC4037403

[B13] Dillhoff M. Liu J. Frankel W. Croce C. Bloomston M. (2008). MicroRNA-21 is overexpressed in pancreatic cancer and a potential predictor of survival. *J. Gastrointest. Surg.* 12 2171–2176. 10.1007/s11605-008-0584-x 18642050 PMC4055565

[B14] Edgar R. Domrachev M. Lash A. E. (2002). Gene expression omnibus: NCBI gene expression and hybridization array data repository. *Nucleic Acids Res.* 30 207–210. 10.1093/nar/30.1.207 11752295 PMC99122

[B15] Feng C. Song C. Ning Z. Ai B. Wang Q. Xu Y. (2019). ce-Subpathway: identification of ceRNA-mediated subpathways via joint power of ceRNAs and pathway topologies. *J. Cell Mol. Med.* 23 967–984. 10.1111/jcmm.13997 30421585 PMC6349186

[B16] Feng C. Zhang J. Li X. Ai B. Han J. Wang Q. (2016). Subpathway-CorSP: identification of metabolic subpathways via integrating expression correlations and topological features between metabolites and genes of interest within pathways. *Sci. Rep.* 6:33262. 10.1038/srep33262 27625019 PMC5021946

[B17] Han J. Han X. Kong Q. Cheng L. (2020). psSubpathway: a software package for flexible identification of phenotype-specific subpathways in cancer progression. *Bioinformatics* 36 2303–2305. 10.1093/bioinformatics/btz894 31821408

[B18] Han J. Liu S. Jiang Y. Xu C. Zheng B. Jiang M. (2018). Inference of patient-specific subpathway activities reveals a functional signature associated with the prognosis of patients with breast cancer. *J. Cell Mol. Med.* 22 4304–4316. 10.1111/jcmm.13720 29971923 PMC6111825

[B19] Hanahan D. Weinberg R. A. (2011). Hallmarks of cancer: the next generation. *Cell* 144 646–674. 10.1016/j.cell.2011.02.013 21376230

[B20] Hsu S. D. Tseng Y. T. Shrestha S. Lin Y. L. Khaleel A. Chou C. H. (2014). miRTarBase update 2014: an information resource for experimentally validated miRNA-target interactions. *Nucleic Acids Res.* 42 D78–D85. 10.1093/nar/gkt1266 24304892 PMC3965058

[B21] Hu Q. L. Jiang Q. Y. Jin X. Shen J. Wang K. Li Y. B. (2013). Cationic microRNA-delivering nanovectors with bifunctional peptides for efficient treatment of PANC-1 xenograft model. *Biomaterials* 34 2265–2276. 10.1016/j.biomaterials.2012.12.016 23298779

[B22] Imamura T. Komatsu S. Ichikawa D. Miyamae M. Okajima W. Ohashi T. (2017). Depleted tumor suppressor miR-107 in plasma relates to tumor progression and is a novel therapeutic target in pancreatic cancer. *Sci. Rep.* 7:e120 10.1016/j.jamcollsurg.2017.07.848 PMC5515843 28720759

[B23] Jiang Q. Wang Y. Hao Y. Juan L. Teng M. Zhang X. (2009). miR2Disease: a manually curated database for microRNA deregulation in human disease. *Nucleic Acids Res.* 37 D98–D104. 18927107 10.1093/nar/gkn714 PMC2686559

[B24] Jiang Y. Qian F. Bai X. Liu Y. Wang Q. Ai B. (2019). SEdb: a comprehensive human super-enhancer database. *Nucleic Acids Res.* 47 D235–D243. 10.1093/nar/gky1025 30371817 PMC6323980

[B25] Judeh T. Johnson C. Kumar A. Zhu D. (2013). TEAK: topology enrichment analysis framework for detecting activated biological subpathways. *Nucleic Acids Res.* 41 1425–1437. 10.1093/nar/gks1299 23268448 PMC3561980

[B26] Lei X. F. Jia S. Z. Ye J. Qiao Y. L. Zhao G. M. Li X. H. (2017). Application values of detection of serum CA199, CA242 and CA50 in the diagnosis of pancreatic cancer. *J. Biolo. Regul. Homeostat. Agents* 31:383. 28685541

[B27] Li C. Han J. Yao Q. Zou C. Xu Y. Zhang C. (2013). Subpathway-GM: identification of metabolic subpathways via joint power of interesting genes and metabolites and their topologies within pathways. *Nucleic Acids Res.* 41:e101. 10.1093/nar/gkt161 23482392 PMC3643575

[B28] Li C. Li X. Miao Y. Wang Q. Jiang W. Xu C. (2009). SubpathwayMiner: a software package for flexible identification of pathways. *Nucleic Acids Res.* 37:e131. 10.1093/nar/gkp667 19706733 PMC2770656

[B29] Li F. Xu Y. Zhang Y. Sun Z. Han J. Zhang C. (2015). Subpathway-GMir: identifying miRNA-mediated metabolic subpathways by integrating condition-specific genes, microRNAs, and pathway topologies. *Oncotarget* 6 39151–39164. 10.18632/oncotarget.5341 26472186 PMC4770763

[B30] Li Q. W. Zhou T. Wang F. Jiang M. Liu C. B. Zhang K. R. (2015). MicroRNA-215 functions as a tumor suppressor and directly targets ZEB2 in human pancreatic cancer. *Genet. Mole. Res.* 14:16133. 10.4238/2015.december.8.2 26662405

[B31] Li H. Hao X. Wang H. Liu Z. He Y. Pu M. (2016). Circular RNA expression profile of pancreatic ductal adenocarcinoma revealed by microarray. *Cell. Physiol.* 40:1334. 10.1159/000453186 27997903

[B32] Li M. Zhao J. Li X. Chen Y. Feng C. Qian F. (2020). HiFreSP: A novel high-frequency sub-pathway mining approach to identify robust prognostic gene signatures. *Brief Bioinform.* 21 1411–1424. 10.1093/bib/bbz078 31350847

[B33] Li Y. Qiu C. Tu J. Geng B. Yang J. Jiang T. (2014). HMDD v2.0: a database for experimentally supported human microRNA and disease associations. *Nucleic Acids Res.* 42 D1070–D1074. 24194601 10.1093/nar/gkt1023 PMC3964961

[B34] Listing H. Mardin W. A. Wohlfromm S. Mees S. T. Haier J. (2015). MiR-23a/-24-induced gene silencing results in mesothelial cell integration of pancreatic cancer. *Br. J. Cancer* 112 131–139. 10.1038/bjc.2014.587 25422915 PMC4453619

[B35] Liu S. Zheng B. Sheng Y. Kong Q. Jiang Y. Yang Y. (2019). Identification of cancer dysfunctional subpathways by integrating DNA methylation, copy number variation, and gene-expression data. *Front. Genet.* 10:441. 10.3389/fgene.2019.00441 31156704 PMC6529853

[B36] Long L. M. Zhan J. K. Wang H. Q. Li S. Chen Y. Y. Liu Y. S. (2017). The clinical significance of miR-34a in pancreatic ductal carcinoma and associated molecular and cellular mechanisms. *Pathobiology* 84 38–48. 10.1159/000447302 27458977

[B37] Loukopoulos P. Shibata T. Katoh H. Kokubu A. Sakamoto M. Yamazaki K. (2007). Genome-wide array-based comparative genomic hybridization analysis of pancreatic adenocarcinoma: identification of genetic indicators that predict patient outcome. *Cancer Sci.* 98 392–400. 10.1111/j.1349-7006.2007.00395.x 17233815 PMC11158398

[B38] Ma C. Nong K. Zhu H. Wang W. Huang X. Yuan Z. (2014). H19 promotes pancreatic cancer metastasis by derepressing let-7’s suppression on its target HMGA2-mediated EMT. *Tumour Biol.* 35 9163–9169. 10.1007/s13277-014-2185-5 24920070

[B39] Masugi Y. Yamazaki K. Emoto K. Effendi K. Tsujikawa H. Kitago M. (2015). Upregulation of integrin β4 promotes epithelial-mesenchymal transition and is a novel prognostic marker in pancreatic ductal adenocarcinoma. *Lab. Invest.* 95 308–319. 10.1038/labinvest.2014.166 25599535

[B40] Nan Y. L. Hu Y. L. Liu Z. K. Duan F. F. Xu Y. Li S. (2016). Relationships between cell cycle pathway gene polymorphisms and risk of hepatocellular carcinoma. *World J. Gastroenterol.* 22 5558–5567. 10.3748/wjg.v22.i24.5558 27350734 PMC4917616

[B41] Ning Z. Feng C. Song C. Liu W. Shang D. Li M. (2019). Topologically inferring active miRNA-mediated subpathways toward precise cancer classification by directed random walk. *Mol. Oncol.* 13 2211–2226. 10.1002/1878-0261.12563 31408573 PMC6763789

[B42] Ogawa N. Inokuchi M. Takagi Y. Sugita H. Kato K. Kojima K. (2015). Clinical significance of platelet derived growth factor-C and -D in gastric cancer. *Oncol. Lett.* 10 3495–3501. 10.3892/ol.2015.3758 26788156 PMC4665846

[B43] Ooi C. H. Oh H. K. Wang H. Z. Tan A. L. Wu J. Lee M. (2011). A densely interconnected genome-wide network of microRNAs and oncogenic pathways revealed using gene expression signatures. *PLoS Genet.* 7:e1002415. 10.1371/journal.pgen.1002415 22194702 PMC3240594

[B44] Park M. Kim M. Hwang D. Park M. Kim W. K. Kim S. K. (2014). Characterization of gene expression and activated signaling pathways in solid-pseudopapillary neoplasm of pancreas. *Modern Pathol. Off. J. U. S.* 27 580–593. 10.1038/modpathol.2013.154 24072181

[B45] Peng Z. Guo Z. Hu R. He X. Jiao X. Zhu X. (2015). Interaction between microRNA-181a and TNFAIP1 regulates pancreatic cancer proliferation and migration. *Tumor Biol.* 36 9693–9701. 10.1007/s13277-015-3704-8 26152285

[B46] Plaisier C. L. Pan M. Baliga N. S. (2012). A miRNA-regulatory network explains how dysregulated miRNAs perturb oncogenic processes across diverse cancers. *Genome Res.* 22:2302. 10.1101/gr.133991.111 22745231 PMC3483559

[B47] Qian F. C. Li X. C. Guo J. C. Zhao J. M. Li Y. Y. Tang Z. D. (2019). SEanalysis: a web tool for super-enhancer associated regulatory analysis. *Nucleic Acids Res.* 47 W248–W255. 10.1093/nar/gkz302 31028388 PMC6602466

[B48] Reid-Lombardo K. M. Fridley B. L. Bamlet W. R. Cunningham J. M. Sarr M. G. Petersen G. M. (2013). Survival is associated with genetic variation in inflammatory pathway genes among patients with resected and unresected pancreatic cancer. *Ann. Surg.* 257 1096–1102. 10.1097/sla.0b013e318275b7e5 23360921 PMC3677709

[B49] Sidiropoulos K. G. White N. M. Bui A. Ding Q. Boulos P. Pampalakis G. (2014). Kallikrein-related peptidase 5 induces miRNA-mediated anti-oncogenic pathways in breast cancer. *Oncoscience* 1 709–724. 10.18632/oncoscience.91 25593998 PMC4278268

[B50] Siegel R. L. Miller K. D. Jemal A. (2017). Cancer statistics, 2017. *CA Cancer J. Clin.* 67 7–30. 10.3322/caac.21387 28055103

[B51] Song B. Zheng K. Ma H. Liu A. Jing W. Shao C. (2015). miR-429 determines poor outcome and inhibits pancreatic ductal adenocarcinoma growth by targeting TBK1. *Cell. Physiol. Biochem.* 35 1846–1856. 10.1159/000373995 25833382

[B52] Thackeray J. T. Pietzsch S. Stapel B. Rickehoch M. Lee C. W. Bankstahl J. P. (2017). Insulin supplementation attenuates cancer-induced cardiomyopathy and slows tumor disease progression. *JCI Insight.* 2:e93098. 10.1172/jci.insight.93098 PMC5436547 28515362

[B53] Tusher V. G. Tibshirani R. Chu G. (2001). Erratum: significance analysis of microarrays applied to the ionizing radiation response. *Proc. Natl. Acad. Sci. U.S.A.* 98 5116–5121. 10.1073/pnas.091062498 11309499 PMC33173

[B54] Tzeng C. W. Frolov A. Frolova N. Jhala N. C. Howard J. H. Vickers S. M. (2007). Pancreatic cancer epidermal growth factor receptor (EGFR) intron 1 polymorphism influences postoperative patient survival and in vitro erlotinib response. *Ann. Surg. Oncol.* 14 2150–2158. 10.1245/s10434-007-9409-5 17453292

[B55] Vergoulis T. Vlachos I. S. Alexiou P. Georgakilas G. Maragkakis M. Reczko M. (2012). TarBase 6.0: capturing the exponential growth of miRNA targets with experimental support. *Nucleic Acids Res.* 40 222–229. 10.1093/nar/gkr1161 PMC3245116 22135297

[B56] Vrahatis A. G. Dimitrakopoulos G. N. Tsakalidis A. K. Bezerianos A. (2015). “Identifying miRNA-mediated signaling subpathways by integrating paired miRNA/mRNA expression data with pathway topology,” in *Proceedings of the 2015 37th Annual International Conference of the IEEE Engineering in Medicine and Biology Society (EMBC)*, Milan. 10.1109/EMBC.2015.7319270 26737170

[B57] Vrahatis A. G. Dimitrakopoulou K. Balomenos P. Tsakalidis A. K. Bezerianos A. (2016). CHRONOS: a time-varying method for microRNA-mediated subpathway enrichment analysis. *Bioinformatics* 32 884–892. 10.1093/bioinformatics/btv673 26568631

[B58] Walters D. M. Lindberg J. M. Adair S. J. Newhook T. E. Cowan C. R. Stokes J. B. (2013). Inhibition of the growth of patient-derived pancreatic cancer xenografts with the MEK inhibitor trametinib is augmented by combined treatment with the epidermal growth factor receptor/HER2 inhibitor lapatinib. *Neoplasia* 15 U143–U207. 10.1593/neo.121712 PMC3579317 23441129

[B59] Wang L. Zhang J. Wan L. Zhou X. Wang Z. Wei W. (2015). Targeting Cdc20 as a novel cancer therapeutic strategy. *Pharmacol. Therapeut.* 151 141–151. 10.1016/j.pharmthera.2015.04.002 25850036 PMC4457591

[B60] Wang W. Zhao L. Wei X. Wang L. Liu S. Yu Y. (2016). MicroRNA-320a promotes 5-FU resistance in human pancreatic cancer cells. *Sci. Rep.* 6:27641. 10.1038/srep27641 PMC4899709 27279541

[B61] Wang Z. Ahmad A. Li Y. Kong D. Azmi A. S. Banerjee S. (2010). Emerging roles of PDGF-D signaling pathway in tumor development and progression. *Biochim. Biophys. Acta* 1806 122–130. 10.1016/j.bbcan.2010.04.003 20434526 PMC2885511

[B62] Wee P. Wang Z. (2017). Epidermal growth factor receptor cell proliferation signaling pathways. *Cancers* 9:52. 10.3390/cancers9050052 28513565 PMC5447962

[B63] Wong D. J. Hurvitz S. A. (2014). Recent advances in the development of anti-HER2 antibodies and antibody-drug conjugates. *Ann. Transl. Med.* 2:122. 10.3978/j.issn.2305-5839.2014.08.13 PMC4260046 25568875

[B64] Xiao F. Zuo Z. Cai G. Kang S. Gao X. Li T. (2009). miRecords: an integrated resource for microRNA-target interactions. *Nucleic Acids Res.* 37 D105–D110. 10.1093/nar/gkn851 18996891 PMC2686554

[B65] Xiao G. Wang X. Yu Y. (2017). CXCR4/Let-7a axis regulates metastasis and chemoresistance of pancreatic cancer cells through targeting HMGA2. *Cell. Physiol. Biochem.* 43 840–851. 10.1159/000481610 28954272

[B66] Xie B. Ding Q. Han H. Wu D. (2013). miRCancer: a microRNA-cancer association database constructed by text mining on literature. *Bioinformatics* 29 638–644. 10.1093/bioinformatics/btt014 23325619

[B67] Xin L. Gao J. Wang D. Lin J. H. Liao Z. Ji J. T. (2017). Novel blood-based microRNA biomarker panel for early diagnosis of chronic pancreatitis. *Sci. Rep.* 7:40019. 10.1038/srep40019 28074846 PMC5225423

[B68] Yamaguchi M. Okamura S. Yamaji T. Iwasaki M. Tsugane S. Shetty V. (2019). Plasma cytokine levels and the presence of colorectal cancer. *PLoS One* 14:e0213602. 10.1371/journal.pone.0213602 30883594 PMC6422333

[B69] Yang J. Zhang H. F. Qin C. F. (2017). MicroRNA-217 functions as a prognosis predictor and inhibits pancreatic cancer cell proliferation and invasion via targeting E2F3. *Eur. Rev. Med. Pharmacol. Sci.* 21 4050–4057. 29028097

[B70] Yao J. Zhang C. Chen Y. Gao S. (2019). Downregulation of circular RNA circ-LDLRAD3 suppresses pancreatic cancer progression through miR-137-3p/PTN axis. *Life Sci.* 239:116871. 10.1016/j.lfs.2019.116871 31521692

[B71] Yu G. Wang L. Han Y. He Q. (2012). clusterProfiler: an R package for comparing biological themes among gene clusters. *OMICS J. Integr. Biol.* 16 284–287. 10.1089/omi.2011.0118 22455463 PMC3339379

[B72] Yu Z. Zhao S. Wang L. Wang J. Zhou J. (2019). miRNA-339-5p plays an important role in invasion and migration of pancreatic cancer cells. *Med. Sci. Monit.* 25 7509–7517. 10.12659/MSM.917038 31588120 PMC6792519

[B73] Zhao L. Sun H. Kong H. Chen Z. Chen B. Zhou M. (2017). The Lncrna-TUG1/EZH2 axis promotes pancreatic cancer cell proliferation, migration and EMT phenotype formation through sponging Mir-382. *Cell. Physiol.* 42:2145. 10.1159/000479990 28813705

