# Supplementary figures and images for: MiRNA-Mediated Subpathway Identification and Network Module Analysis to Reveal Prognostic Markers in Human Pancreatic Cancer
Source: Front Genet. 2020 Dec 9;11:606940. doi: 10.3389/fgene.2020.606940 (PMC7756031; doi:10.3389/fgene.2020.606940)

Figure S1

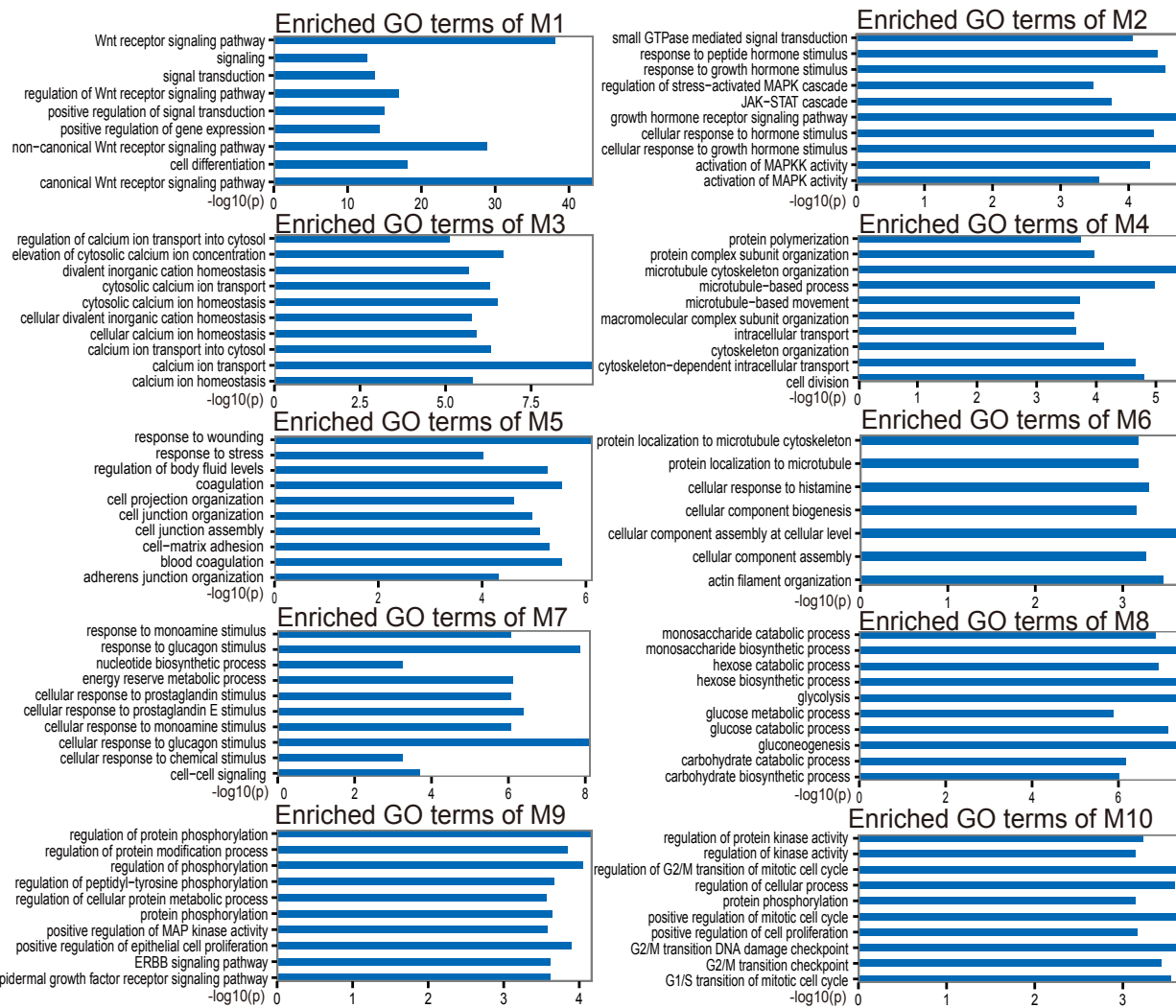

Supplement: Supplementary Figure 1 — The enriched GO terms for miRNA-mRNA modules. [file Data_Sheet_1.PDF]

**Figure S2**

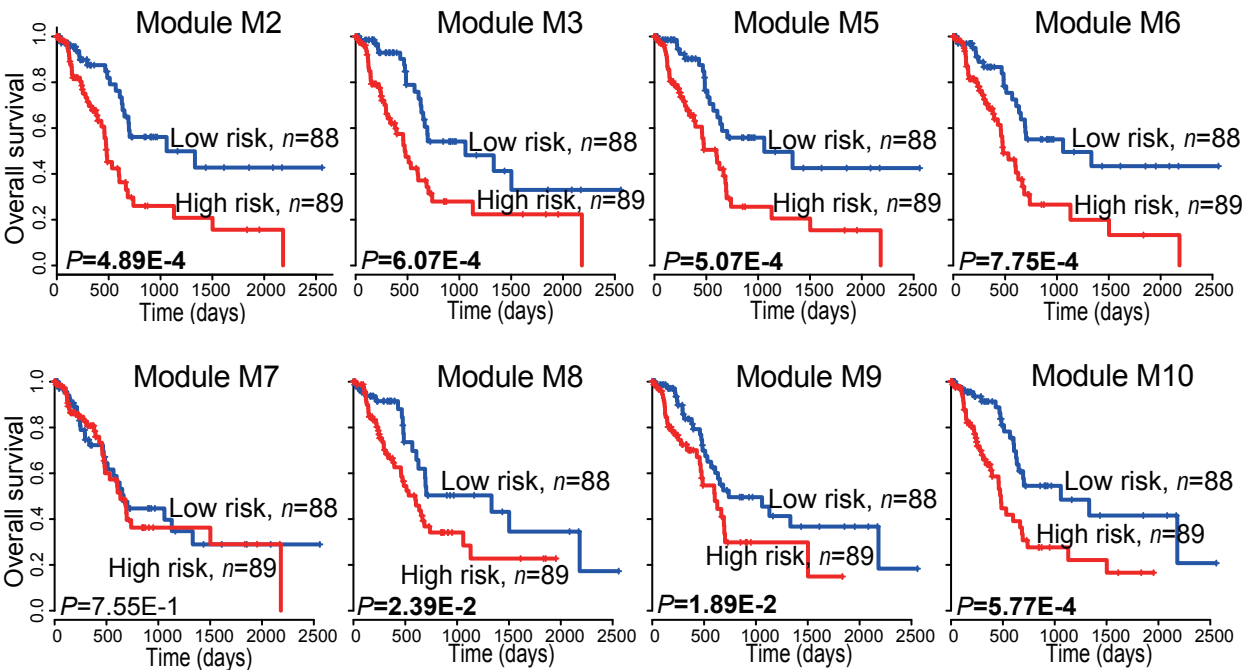

Supplement: Supplementary Figure 2 — Survival analysis for miRNA-mRNA modules based on genes. [file Data_Sheet_2.PDF]

**Figure S3**

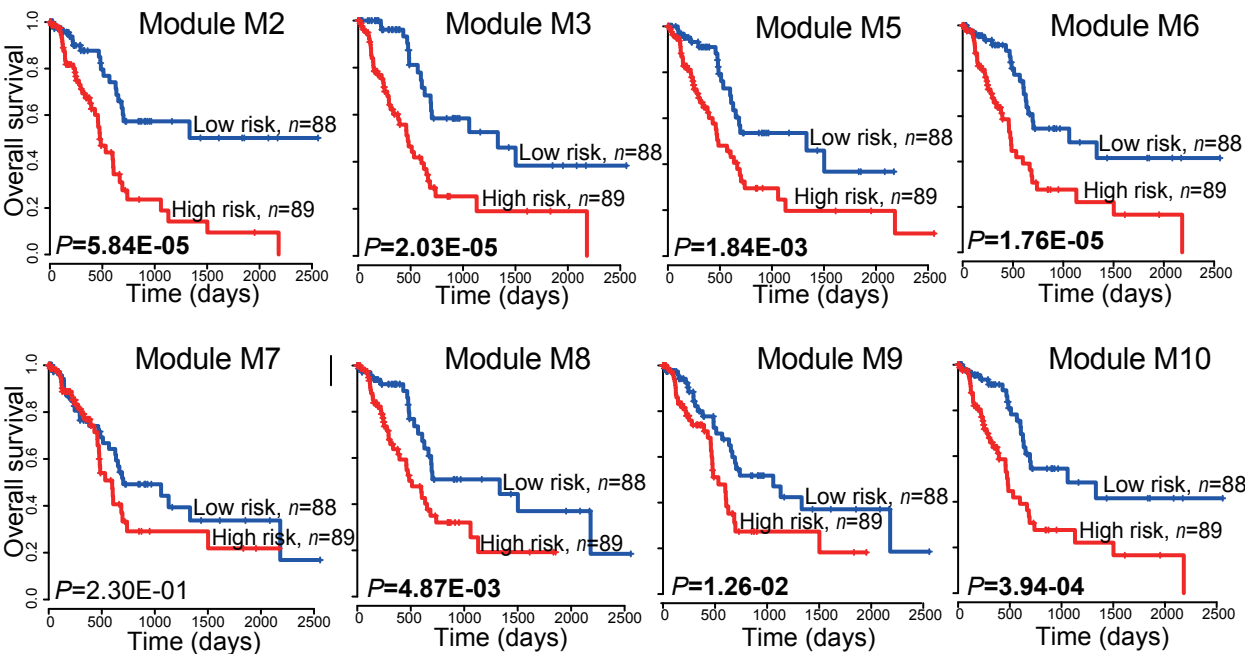

Supplement: Supplementary Figure 3 — Survival analyses for miRNA-mRNA modules based on miRNAs and genes. [file Data_Sheet_3.PDF]
